# Molecular Disruption of Ion Transport Peptide Receptor Results in Impaired Water Homeostasis and Developmental Defects in *Bombyx mori*

**DOI:** 10.3389/fphys.2020.00424

**Published:** 2020-05-20

**Authors:** Lili Sun, Zhongjie Zhang, Ru Zhang, Ye Yu, Fangying Yang, Anjiang Tan

**Affiliations:** ^1^College of Forestry, Northeast Forestry University, Harbin, China; ^2^CAS Center for Excellence in Biotic Interactions, University of Chinese Academy of Sciences, Beijing, China; ^3^Key Laboratory of Insect Developmental and Evolutionary Biology, CAS Center for Excellence in Molecular Plant Sciences, Shanghai Institute of Plant Physiology and Ecology, Chinese Academy of Sciences, Shanghai, China

**Keywords:** *Bombyx mori*, ion transport peptide receptor, CRISPR/Cas9, water homeostasis, development

## Abstract

Insect ion transport peptides (ITPs) are important regulators of many physiological processes and they exert their functions by interacting with their receptors (ITPRs). In the current study, we comprehensively investigated the physiological functions of ITPR in the lepidopteran model insect, the silkworm (*Bombyx mori*), using the clustered regularly interspaced short palindromic repeats (CRISPR)/CRISPR-associated protein-9 nuclease (Cas9) genome editing technique. Mutations in silkworm ITPR (*BNGR-A2*) resulted in a prolongnation of the larval stage by 3.5-day as well as failure in wing expansion of moths. The *BNGR-A2* mutation accelerated food transition throughout the digestive tract, which is 1.55-fold that of wild type (WT) insects. Excretion was 1.56-fold of WT insects during the larval stage, resulting in the loss of body water content. Loss of *BNGR-A2* function induced significant upregulation of nitric oxide synthase (NOS) enzyme activity and nitric oxide (NO) content, as well as downstream Ca^2+^/NO/cGMP signaling pathways. Key genes in insulin and ecdysone signaling pathways were also affected by *BNGR-A2* disruption. Our data show that ITPR plays key roles in regulating insect water homeostasis and development.

## Introduction

Ion transport peptides (ITPs) and its alternatively spliced homologous ITP-like (ITPL) products belong to the crustacean hyperglycemic hormone (CHH) family of peptides. They are homologs of the crustacean hyperglycemic hormones of molting-inhibiting hormone (MIH) and gonad/vitellogenesis-inhibiting hormone (GIH) (Audsley et al., 2006; Webster et al., 2012). In crustaceans, CHH family peptides play roles in energy metabolism, molting, reproduction, immune defense, and osmotic regulation, as well as in homeostatic regulation of stress responses (Sonobe et al., 2001; Webster et al., 2012). In insects, ITP and ITPL with Cl^–^, Na^+^, and Ca^2+^ transport, as well as fluid reabsorption functions, have been identified (Audsley et al., 1992; Drexler et al., 2007; Dircksen et al., 2008; Webster et al., 2012; Nagai et al., 2014; Yu et al., 2016). In *Drosophila melanogaster*, ITP plays a critical role in development and also participates in the control of locomotor rhythms (Johard et al., 2009; Hermann-Luibl et al., 2014; Gáliková et al., 2018), and the mutation of ITP is embryonically lethal (Park et al., 2004). Gáliková et al. (2018) work identified master regulatory roles of ITP in water homeostasis of *Drosophila*; ITP levels increase under desiccation stress and protect the fly from water loss by increasing thirst, reducing excretion rate, and promoting ingestion of water instead of food. Several studies have shown that ITPs function during ecdysis in *Manduca sexta* (Drexler et al., 2007). The insect ITPL also participates in ovarian maturation in *Tribolium castaneum* (Begum et al., 2009) and regulates wing expansion and cuticle melanism in *Nilaparvata lugens* (Yu et al., 2016).

Insect ITP and ITPL act through their receptors (ITPRs) to function in many physiological processes. However, no ITPRs have been identified in the model insect *D. melanogaster* thus far (Gáliková et al., 2018; Nässel and Zandawala, 2019). In the lepidopteran model insect, *Bombyx mori*, it has been reported that two *Bombyx* neuropeptide G protein-coupled receptors (BNGRs), BNGR-A2 and BNGR-A34, are native receptors for ITP (Nagai et al., 2014). The ITP receptor, BNGR-A34, is conserved across insect species, whereas BNGR-A2 may have a species-specific role in lepidopterans (Nagai et al., 2014). Although a positive correlation between response of the receptor and bioactivity of the ligand has been verified *in vitro* (Nagai et al., 2014), the physiological functions of both ITP/ITPR and their receptors in insects *in vivo* remain to be elucidated.

In this study, we investigated the physiological functions of BNGR-A2 using the CRISPR/Cas9 genome editing technique. Loss of *BNGR-A2* function extended the larval developmental stage and caused failure of wing expansion in moths. The *BNGR-A2* mutation also accelerated food transition throughout the digestive tract as well as excretion. Our data therefore reveals for the first time that ITPR plays critical roles in regulating water homeostasis and developement in *B. mori*.

## Materials and Methods

### Silkworm Strains

A multivoltine silkworm strain, Nistari, was used in all experiments. Larvae were reared with fresh mulberry leaves at 25°C and 75% relative humidity (RH) (Tan et al., 2013).

### Plasmid Construction and Germline Transformation

The activator line of *pBac* [*IE1-EGFP-Nos-Cas9*] (*Nos-Cas9*) was used to construct the transgenic CRISPR/Cas9 system. Cas9 was driven by the germ-cell-specific promoter, *Nos*, as described previously (Xu et al., 2017b). The effector line *pBac* [IE1-DsRed × 2-U6-2 × *BNGR-A2-*sgRNAs] (*BNGR-A2*-sgRNAs) was under the control of the silkworm small nuclear RNA promoter U6. The plasmids targeting *BNGR-A2* were constructed through a series of cloning steps (Xu et al., 2017a); primer sequences are listed in Supplementary Table S1.

For silkworm germline transformation, G0 Nistari embryos within 8 h of oviposition were separately injected with a plasmid mixture of *Nos-Cas9* or *BNGR-A2-sgRNAs*. Hatched larvae were reared to the adult stage and G0 moths were sib-mated or backcrossed with wild-type (WT) moths. Using a fluorescence microscope (Nikon AZ100), G1 offspring were screened for the marker gene during the embryonic stage. The *Nos-Cas9* line and the *BNGR-A2*-sgRNA line were crossed to produce heterozygous F1 progeny (mutant was obtained by indicator of two-color fluorescence), which was then used in subsequent experiments.

### Genomic DNA Extraction and Mutagenesis Analysis

The *BNGR-A2* mutant genomic DNA was extracted using a nucleic acid isolation system DP323 (TianGen, Beijing, China), and the DNA fragments, including the designed sgRNA targeting site, were amplified using EasyTaq^®^ DNA Polymerase (TransGen Biotech, Beijing, China). The PCR conditions were as follows: 94°C for 5 min, 35 cycles at 94°C for 30 s, 60°C for 30 s, and 72°C for 1 min, followed by a final extension period of 72°C for 10 min. The PCR products were cloned into pMD19-T-Simple (Takara, Dalian, China) and subsequently sequenced. The primers that were designed to detect mutagenesis in targeted sites are listed in Supplementary Table S1.

### Bioassays

The cumulative numbers of fecal pellets from 20 fifth instar silkworms (0 day of fifth instar larvae, L5D0) were counted in the breeding box every hour. Droppings were counted in three replicates. The statistical significance was analyzed by two-way ANOVA, and the number of fresh fecal masses that were deposited within 2 h (the given period) was used.

To measure water content, L5D0 silkworm larvae were dehydrated at 80°C until a constant weight. The weight of 10 larvae was recorded before and after dehyration using a Mettler MT5 analytical microbalance (Columbus, OH, United States). The difference in water content between the fresh and dry weights was calculated and is expressed as the percent of the fresh body weight. Three replicates (each consisting of 10 silkworms) were tested per WT and mutant silkworm.

### Ecdysteroid Titer and NOS Activity Determination

Haemolymph was extracted from transgenic or WT larvae (L5D3). Total ecdysteroids in the haemolymph samples were extracted as previously described (Mirth et al., 2005). Concentrations of total ecdysteroids were quantified using a 20-Hydroxyecdysone EIA kit (Cayman Chemicals, Ann Arbor, MI, United States) according to the manufacturer’s instructions.

The nitric oxide synthase (NOS) kit (Nanjing Jiancheng Bioengineering Institute, Nanjing, China) was used to detect the total NOS activity in silkworm hindgut. The total nitric oxide (NO) was determined using a Nitric Oxide Assay Kit (Beyotime, Shanghai, China). Protein concentrations were measured using the Bradford Protein Assay Kit (Beyotime, Shanghai, China). Each measurement was from at least four biological replicates including two males and two females. Data were normalized to the protein concentration.

### RNA Isolation, cDNA Synthesis, and Quantitative Real-Time PCR (qRT-PCR) Analysis

Total RNA was extracted from the fat body and hindgut of L5D3 animals using Trizol reagent (Invitrogen, Carlsbad, CA, United States) in accordance with the manufacturer’s instructions. Then, 0.5 μg of RNA was reverse-transcribed for cDNA using TransScript^®^ RT/RI Enzyme Mix (TransGen Biotech, Beijing, China). qRT-PCR was performed using a SYBR Green PCR Kit (Toyobo, Osaka, Japan) and an ABI Stepone plus real-time PCR system (Applied Biosystems, United States). Each 20 μL reaction contained 10 μL of SYBR Green real-time PCR master mix (Toyobo, Osaka, Japan), 0.5 μM of each forward and reverse primer, and 2 μL of cDNA template (equivalent to 100 ng of total RNA). Primers used for amplification are listed in Supplementary Table S1. Thermal cycling parameters were 94°C for 5 min followed by 40 cycles at 94°C for 30 s and 60°C for 1 min. For each sample, a melting curve was generated at the end of each run to allow assessment of product purity. Expression levels were calculated according to the 2^–ΔΔ*CT*^ method (Pfaffl et al., 2002), and relative expression levels were calculated by dividing mutant transcription levels by levels recorded in wild type, WT insects.

### RNA-Seq Analysis

Hindguts from WT and mutant L5D3 animals were separately collected and used to construct a transcriptome library. Each type of sample was collected from four animals (two males and two females). The cDNA libraries were sequenced using an Illumina BGISEQ-500 platform (BGI, Wuhan, China). Total RNA was isolated as described previously, and cDNA libraries were prepared according to Illumina’s protocol. The raw data were filtered using Tophat software, corrected, and mapped to the silkworm genome sequence. The total reading was normalized by a plurality of normalization factors. Transcript levels were calculated using standard readings per petaflop of mapping readings. The difference between the mutant and the control groups is represented by the *P* value and fold change (Δ*BNGR-A2*/WT). Differentially expressed genes with a significance level of *P* < 0.001 and fold change (Δ*BNGR-A2*/WT) > 2 were enriched in each comparison.

### Statistical Analyses

Two-tailed Student’s *t*-test and one-way ANOVA were used to analyze measurement variables. Asterisk symbols reflect significant differences (^∗^*P* < 0.05, ^∗∗^*P* < 0.01, ^∗∗∗^*P* < 0.001, ^****^P < 0.0001). Error bars represent SEM. Measurement variable data was analyzed using Excel Workbook (version 2016) and data related to nominal variables were analyzed by GraphPad Prism (version 7.0).

## Results

### Generation of *BNGR-A2* Targeted Mutations Using the Transgenic CRISPR/Cas9 System

**FIGURE 1:**
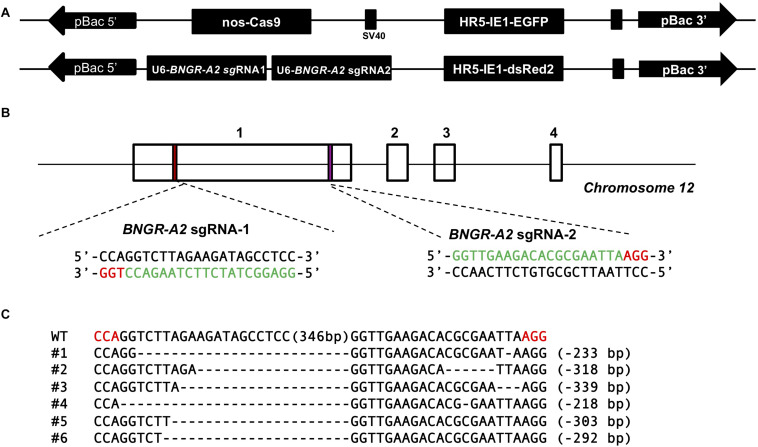
Schematic diagram of *BNGR-A2* sgRNA targeting sites and genomic mutagenesis induced by the transgenic CRISPR/Cas9 system. **(A)** Binary transgenic CRISPR/Cas9 system. **(B)** Schematic diagram of sgRNA-targeting sites. The boxes indicate the four exons of *BNGR-A2*, and the black line represents the gene locus. The sgRNA-targeting sites, *BNGR-A2* sgRNA-1 and *BNGR-A2* sgRNA-2, are located of the exon 1. The sgRNA targeting sequence is in green, and the protospacer adjacent motif (PAM) sequence is in red. **(C)** Various deletion mutageneses were detected in heterozygous *Nos-Cas9: BNGR-A2-sgRNAs* (△*BNGR-A2*) offspring. The dashes in every sequence line represent deleted residues and the detailed indel size is shown on the right. The numbers in brackets in the middle of each sequence refer to the 349 bp interspace fragment that was found between the targeting sites. The red sequence indicates the PAM sequence.

A binary transgenic CRISPR/Cas9 system (Li et al., 2015) was used to generate somatic *BNGR-A2* mutants. This system included two transgenic silkworm lines. One line expressed Cas9 under the control of Nos promoter and the other line expressed two sequence-specific sgRNAs under the control of U6 promoter to target *BNGR-A2* exon 1 (Figures 1A,B). Transgenic animals carrying nos-Cas9 or U6-*BNGR-A2*–sgRNA were fully viable and fertile, indicating that the accumulation of neither Cas9 nor *BNGR-A2*-sgRNA had deleterious effects on silkworm physiology. The offspring crossed by Nos-Cas9 and U6-*BNGR-A2*-sgRNA lines specifically expressed the active Cas9-sgRNA complex in the germ line. The mutations were located at the targeted genomic loci of the *BNGR-A2* gene (Figure 1B). To assess mutation efficiency, five newly hatched silkworms with both red and green fluoresence were randomly selected to extract the genomic DNA. The DNA samples were run through PCR amplification. PCR-amplified fragments were subcloned and sequenced. The results showed that various deletion mutations located at two sgRNA targets and deletions ranged from 215 to 349 bp, indicating that successful mutations were produced using the transgenic CRISPR/Cas9 system. Moreover, the *BNGR-A2* gene was successfully disrupted (Figure 1C).

### Water Content and Excretion Rate of *BNGR-A2* Mutant Larvae

**FIGURE 2:**
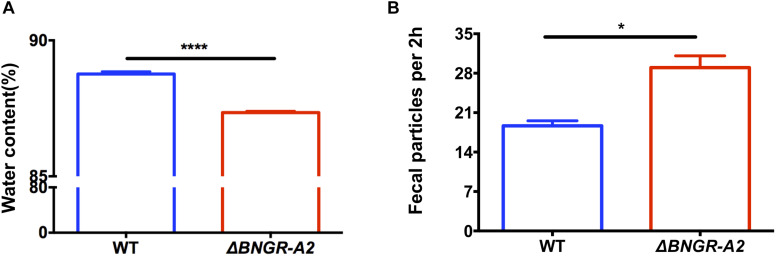
ITP regulates water content, the speed of transit through the digestive tract and the number of defecation events. **(A)** *BNGR-A2* regulates water content. Two-tailed Student’s *t*-test: *P* < 0.0001. **(B)** *BNGR-A2*-loss increases the defecation rate.

Previous studies showed that ITP has a positive effect on the water balance in *Drosophila* (Gáliková et al., 2018). However, whether ITPR affects the water balance in insects is unclear. In order to investigate whether *BNGR-A2* is involved in water homeostasis in *B. mori*, the water contents of WT and mutant 5th instar larvae were determined. The results showed that the water contents of Δ*BNGR-A2* animals was (87.34 ± 0.03)%, which was significantly lower than that of WT animals (88.76 ± 0.05)%; *P* < 0.0001), indicating that the loss of *BNGR-A2* function affected the water homeostasis regulation in *B. mori* (Figure 2A).

In insects, the primary urete enters the hindgut, and water that is not reabsorbed by the posterior intestinal epithelium is usually excreted in the same way as feces (Gäde, 2004). We investigated whether *BNGR-A2* knockout affects excretion. The excretion rate of the 5th instar larvae was calculated by counting the number of fecal particles produced every 2 h. The results showed that each mutant larvae produced 29 fecal particles per 2 h period, which was significantly higher than in WT animals (18.67 fecal particles per 2 h, *P* < 0.05). Loss of *BNGR-A2* function accelerated food transition throughout the digestive tract (Figure 2B), resulting in more frequent defecation events.

### Phenotypic Defects in *BNGR-A2* Mutants

**FIGURE 3:**
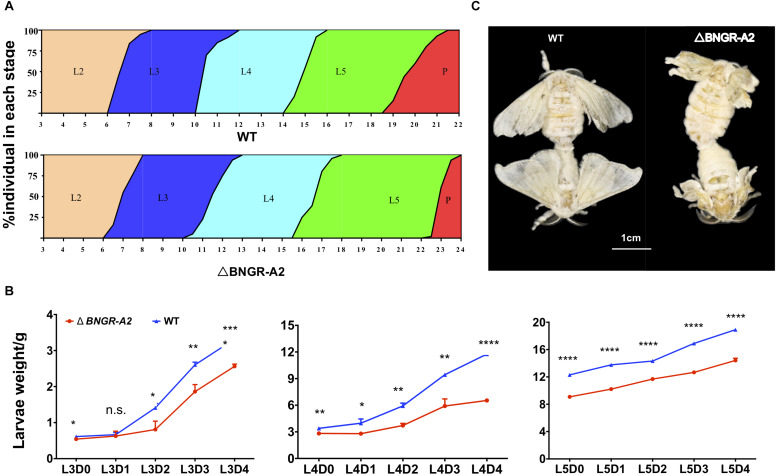
*BNGR-A2* gene mutation affects larval growth and development. **(A)** The stages of larval and pupal development in WT and △*BNGR-A2* (L2–L5 represent 2nd to 5th instar, respectively; P, pupae). **(B)** The L3, L4, and L5 weight of WT and △*BNGR-A2* (*n* = 3 samples per treatment, 10 animals/sample). **(C)** Wing expansion failed in △*BNGR-A2* insects. The left graph shows the △*BNGR-A2* insects, the right graph shows the WT insects.

The *BNGR-A2* mutant has no deleterious phenotype during embryonic development. From the end of the 2nd larval instar, mutant larvae exhibited a significant delay in growth rate relative to WT animals (Figures 3A,B). Compared with WT animals, the development of mutant larvae was delayed by half a day during the 3rd larval stage. The weight of mutant larvae was 57.41% of WT animals at L3D2. During the 4th instar larval stage, the weight of the mutant larvae was 55.58% that of WT animals, and development was delayed by 2.5 days. The weight of the 5th instar mutant larvae was 73.84% of the WT animals at L5D0, and the weight of the L5D4 mutant was similar to that of the L5D2 WT animals (Figures 3A,B). The half pupation time (PT50) of the mutant silkworm was delayed by 3 days relative to WT silkworms (Figure 3A). Pupal weight did not show significant differences between mutant and WT animals. After eclosion, the fore and hindwings of mutant moths became crumpled (Figure 3C).

### Ca^2+^/NO/cGMP Signaling in *BNGR-A2* Mutants

**FIGURE 4:**
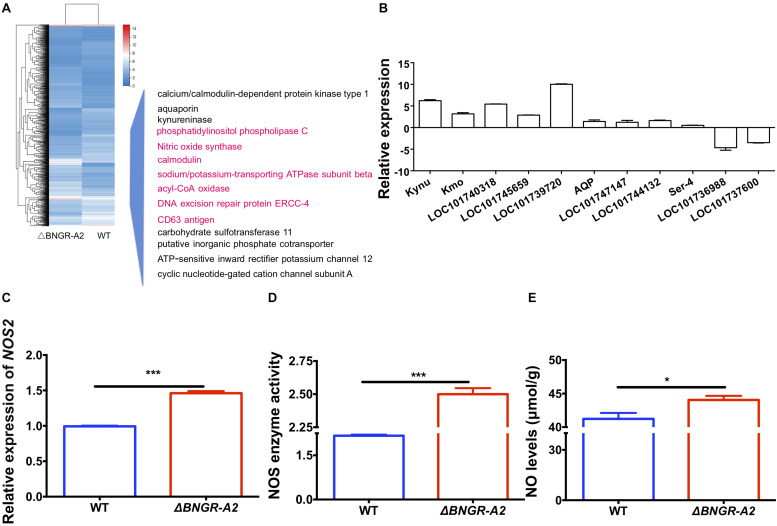
Cluster analysis of RNA-Seq data leads to the identification of Ca^2+^/NO/cGMP as downstream of the *BNGR-A2* pathway. **(A)** Cluster analysis of differentially expressed genes (DEGs) in the transcriptome (in hindguts, HG). Several important genes (highlighted pink) were involved in Ca^2+^/NO/cGMP signaling and display expression patterns after the loss of *BNGR-A2* transcription levels. Logarithmic fold alteration of △*BNGR-A2* versus WT is shown in the heat map. Red and blue colors indicate up- and down-regulation, respectively. **(B)** Transcriptome verification of genes involved in water transport, Log2. **(C)** Change in the expression of *NOS2* genes in HG (*n* = three samples per treatment, four (two males and two females) silkworms/sample). **(D)** Enzyme activity change of NOS in HG (*n* = three samples per treatment, four (two males and two females) silkworms/sample). **(E)** Changes in the levels of NO in HG [*n* = two samples per treatment, four (two males and two females) silkworms/sample].

To explore the molecular mechanisms underlying defective phenotypes induced by *BNGR-A2* mutations, we perfomed RNA-seq analysis by using hindgut tissues between WT and mutant larvae. A total of 1,288 differentially expressed genes (612 up-regulated and 676 down-regulated) were identified (Figure 4A and Supplementary Figure 1SA). The up- and down-regulated genes were allocated into three categories according to the Gene Ontology (GO) terms: molecular function, cellular component, and biological process (Supplementary Figure 1SB). Among GO categories, the “catalytic activity” in “molecular function,” “membrane” and “membrane part” in “cellular component,” and “metabolic process” in “biological process” were the most abundant protein classes. Kyoto Encyclopedia of Genes and Genomes (KEGG) enrichment analysis revealed that water-transport-related pathways, such as the calcium signaling pathway, cGMP-PKG signaling pathway and cAMP signaling pathway, were annotated (Table 1 and Supplementary Figure 1SC).

**TABLE 1:** Annotated DEGs of Ca^2+^/NO/cAMP signaling pathway.

Signaling pathway	Function annotation	*P* value	Gene ID	log2 (ΔBNGR-A2/WT)	Nr
	GPCR	0	NGR-A24	3.36	NP_001127722.1| 3.9e-225| neuropeptide receptor A24 [*Bombyx mori*]
	CALM	0	LOC101742890	2.94	XP_004927631.1| 4.9e-135| neo-calmodulin [*Bombyx mori*]
	GPCR	3.24E-21	NGR-A17	1.66	NP_001127715.1| 7.0e-204| neuropeptide receptor A17 [*Bombyx mori*]
	NOS	5.22E-26	NOS2	1.44	XP_012545275.1| 0.0e+00| nitric oxide synthase-like protein isoform X1 [*Bombyx mori*]
	GPCR	3.45E-07	NGR-A32	1.17	NP_001127748.1| 1.2e-215| neuropeptide receptor A32 [*Bombyx mori*]
	GPCR	4.10E-131	LOC101736629	–1.04	XP 022130216.1| 0.0e+00| cytosolic carboxypeptidase 4-like isoform X6 *[Pieris rapae]*
Calcium	ROC	6.28E-16	BGI novel G000814	–1.23	
signaling pathway	SERCA	2.54E-13	LOC101735353	–1.28	XP_021207909.1| 6.5e-220| uncharacterized protein LOC101735353 [*Bombyx mori*]
	CALM	7.40E-08	LOC101741700	–1.28	XP_004922728.1| 1.3e-163| sarcoplasmic calcium-binding protein [*Bombyx mori*]
	SPHK	1.76E-90	LOC101742466	–1.31	XP_004931675.2| 0.0e+00| sphingosine kinase 1 [*Bombyx mori*]
	GPCR	1.82E-05	LOC101737969	–1.55	XP_012551041.1| 3.2e-262| beta-3 adrenergic receptor [*Bombyx mori*]
	IP3 3K	2.91E-192	LOC101739177	–1.83	XP_012547652.1| 1.2e-260| uncharacterized protein LOC101739177 [*Bombyx mori*]
	PLC β	0.000261562	LOC101743946	–2.50	XP_021206545.1| 0.0e+00| uncharacterized protein LOC101743946 isoform X1 [*Bombyx mori*]
	GPCR	1.07E-22	LOC101743833	–2.94	XP_021204873.1| 0.0e+00| cytosolic carboxypeptidase 2 [*Bombyx mori*]
cGMP-PKG	CAM	0	LOC101742890	2.94	XP_004927631.1| 4.9e-135| neo-calmodulin [*Bombyx mori*]
signaling pathway	ATPase	1.03E-108	LOC101746012	1.21	XP_004929151.1| 1.3e-193| sodium/potassium-transporting ATPase subunit beta-2 [*Bombyx mori*]
	INS	3.57E-08	BmILP	1.15	XP_012548888.1| 1.1e-66| insulin-like peptide isoform X1 [*Bombyx mori*]
	ROCK	6.10E-07	BGI novel G000435	–1.01	
	ROCK	0.000248524	BGI novel G000569	–1.09	
	CREB	6.19E-07	BGI novel G000948	–1.19	
	SERCA	2.54E-13	LOC101735353	–1.28	XP_021207909.1| 6.5e-220| uncharacterized protein LOC101735353 [*Bombyx mori*]
	CAM	7.40E-08	LOC101741700	–1.28	XP_004922728.1| 1.3e-163| sarcoplasmic calcium-binding protein [*Bombyx mori*]
	ROCK	9.43E-05	BGI novel G000712	–1.32	
	ROCK	2.33E-07	LOC105843016	–1.43	XP_011555716.1| 1.9e-112| PREDICTED: uncharacterized protein LOC105386785 [*Plutella xylostella*]
	SRF	7.66E-32	LOC101744587	–1.62	XP_012552250.1| 8.2e-88| serum response factor homolog [*Bombyx mori*]
	GATA-4	6.67E-33	LOC101737855	–2.36	XP_004924467.1| 0.0e+00| transcription factor GATA-6 isoform X1 [*Bombyx mori*]
	PLC β	0.000261562	LOC101743946	–2.50	XP_021206545.1| 0.0e+00| uncharacterized protein LOC101743946 isoform X1 [*Bombyx mori*]
	CNG	2.99E-44	LOC101738542	–3.71	XP_004931049.1| 0.0e+00| cyclic nucleotide-gated cation channel beta-1 isoform X2 [*Bombyx mori*]
cAMP	CAM	0	LOC101742890	2.94	XP_004927631.1| 4.9e-135| neo-calmodulin [*Bombyx mori*]
signaling pathway	PI3K	2.29E-05	BGI novel G000178	2.57	
	GPCR	3.88E-09	Ser-4	2.32	NP_001037502.1| 1.1e-251| 5-hydroxytryptamine receptor [*Bombyx mori*]
	ACO	0.000104462	LOC101746636	2.12	XP_021206441.1| 0.0e+00| LOW QUALITY PROTEIN: glutamate receptor ionotropic, delta-2 [*Bombyx mori*]
	ATP	1.03E-108	LOC101746012	1.21	XP_004929151.1| 1.3e-193| sodium/potassium-transporting ATPase subunit beta-2 [*Bombyx mori*]
	ROCK	6.10E-07	BGI novel G000435	–1.01	
	GPCR	4.10E-131	LOC101736629	–1.04	XP 022130216.1| 0.0e+00| cytosolic carboxypeptidase 4-like isoform X6 *[Pieris rapae]*
	ROCK	0.000248524	BGI novel G000569	–1.09	
	GPCR	1.33E-12	LOC101745640	–1.10	XP_004934259.1| 5.7e-94| piggyBac transposable element-derived protein 4-like, partial [*Bombyx mori*]
	NMDAE	6.28E-16	BGI novel G000814	–1.23	
	CAM	7.40E-08	LOC101741700	–1.28	XP_004922728.1| 1.3e-163| sarcoplasmic calcium-binding protein [*Bombyx mori*]
	ROCK	9.43E-05	BGI novel G000712	–1.32	
	GPCR	0.000327112	LOC105842734	–1.35	XP_004923418.1| 4.7e-85| piggyBac transposable element-derived protein 4 [*Bombyx mori*]
	ROCK	2.33E-07	LOC105843016	–1.43	XP_011555716.1| 1.9e-112| PREDICTED: uncharacterized protein LOC105386785 [*Plutella xylostella*]
	GPCR	2.01E-38	LOC101739439	–1.81	XP_004923418.1| 0.0e+00| piggyBac transposable element-derived protein 4 [*Bombyx mori*]
	GPCR	1.07E-22	LOC101743833	–2.94	XP_021204873.1| 0.0e+00| cytosolic carboxypeptidase 2 [*Bombyx mori*]
	CNGC	2.99E-44	LOC101738542	–3.71	XP_004931049.1| 0.0e+00| cyclic nucleotide-gated cation channel beta-1 isoform X2 [*Bombyx mori*]

Among these differentially expressed genes (DEGs), several classes of genes and inorganic salt ion transporters, ATP-sensitive inward rectifier potassium channels, uridine nucleosides, potassium voltage-gated channel proteins, calcium/calmodulin-dependent proteins, solute carriers, and organic anions were identified. A large of DEGs were associated with transporters, aquaporins, and water transport genes were heavily annotated, and a class of important signaling molecular genes were annotated. Using qRT-PCR, several types of genes were confirmed in two comparisons (Figure 4A and Table 2), showing elevated transcriptional levels in the hindgut of mutants. The expression patterns of these genes, including inorganic salt ion transporters, calcium/calmodulin-dependent proteins, kynureninase, aquaporins and NOS, were confirmed in mutants and WT comparisons (Figure 4B and Table 2).

**TABLE 2:** Differentially expressed genes associated with water transport in △*BNGR-A2* and WT animals.

Other gene ID	log2 (ΔBNGR-A2/WT)	FDR	*P*-value	Nr
aqp	1.319	1.46E-32	1.26E-33	NP_001106228.1| 2.0e-146| aquaporin [*Bombyx mori*]
Kin	2.424	2.23E-15	3.19E-16	NP_001124363.1| 1.1e-195| leucokinin precursor [*Bombyx mori*]
Kynu	6.640	0	0	NP_001136399.1| 8.9e-254| kynureninase [*Bombyx mori*]
LOC101735517	–1.541	4.32E-11	7.54E-12	XP_012547671.1| 0.0e+00| potassium voltage-gated channel protein eag isoform X3 [*Bombyx mori*]
LOC101735871	3.810	3.03E-172	7.47E-174	XP_012546046.1| 0.0e+00| sodium-independent sulfate anion transporter [*Bombyx mori*]
LOC101736269	–1.022	2.58E-42	1.91E-43	XP_004926573.1| 1.9e-274| synaptic vesicle glycoprotein 2C isoform X1 [*Bombyx mori*]
LOC101736763	2.356	5.14E-16	7.17E-17	XP_012546620.1| 5.9e-287| putative transporter SVOPL [*Bombyx mori*]
LOC101736901	2.158	7.24E-15	1.06E-15	XP_004931037.1| 1.5e-182| carbohydrate sulfotransferase 11 [*Bombyx mori*]
LOC101736988	–2.963	5.36E-50	3.51E-51	XP_004929740.2| 0.0e+00| bumetanide-sensitive sodium-(potassium)-chloride cotransporter isoform X1 [*Bombyx mori*]
LOC101737579	2.670	3.21E-127	1.04E-128	XP_012545206.1| 1.1e-287| putative inorganic phosphate cotransporter [*Bombyx mori*]
LOC101737870	1.853	2.06E-62	1.15E-63	XP_004929455.1| 2.0e-285| putative transporter SVOPL isoform X1 [*Bombyx mori*]
LOC101738222	9.488	3.08E-122	1.03E-123	XP_004930039.1| 9.8e-305| monocarboxylate transporter 9 isoform X1 [*Bombyx mori*]
LOC101738542	–3.709	4.13E-43	2.99E-44	XP_004931049.1| 0.0e+00| cyclic nucleotide-gated cation channel beta-1 isoform X2 [*Bombyx mori*]
LOC101738624	1.857	1.50E-06	3.64E-07	XP_004930042.1| 7.0e-151| trypsin, alkaline C [*Bombyx mori*]
LOC101738703	8.778	5.04E-62	2.85E-63	XP_004925899.1| 7.9e-308| putative inorganic phosphate cotransporter [*Bombyx mori*]
LOC101739382	6.672	2.17E-16	2.97E-17	XP_004934350.2| 0.0e+00| oxysterol-binding protein-related protein 1-like [*Bombyx mori*]
LOC101739498	–3.149	0	0	XP_004925267.1| 4.6e-294| synaptic vesicle glycoprotein 2A [*Bombyx mori*]
LOC101739574	–1.029	2.17E-38	1.70E-39	XP_021209181.1| 1.5e-239| adenosine 3’-phospho 5’-phosphosulfate transporter 1 isoform X2 [*Bombyx mori*]
LOC101739720	10.663	1.41E-208	2.96E-210	XP_021209245.1| 6.8e-298| putative inorganic phosphate cotransporter [*Bombyx mori*]
LOC101739734	1.817	2.74E-05	7.50E-06	XP_021204132.1| 3.4e-245| glutamate receptor 2 isoform X1 [*Bombyx mori*]
LOC101739835	1.203	1.02E-24	1.07E-25	XP_012548689.2| 3.7e-310| sodium/hydrogen exchanger 9B2 isoform X2 [*Bombyx mori*]
LOC101739998	4.450	2.17E-27	2.12E-28	XP_004924083.1| 3.6e-222| ATP-sensitive inward rectifier potassium channel 12 [*Bombyx mori*]
LOC101740153	1.241	8.00E-05	2.32E-05	XP_012548956.1| 0.0e+00| cyclic nucleotide-gated cation channel subunit A [*Bombyx mori*]
LOC101740318	4.555	5.66E-17	7.57E-18	XP_012545113.2| 2.1e-181| synaptic vesicle glycoprotein 2B [*Bombyx mori*]
LOC101740880	1.345	3.43E-30	3.14E-31	XP_012549174.2| 0.0e+00| potassium voltage-gated channel subfamily KQT member 1 isoform X3 [*Bombyx mori*]
LOC101741058	–1.150	0	0	XP_004925076.1| 0.0e+00| ATP-dependent Clp protease ATP-binding subunit clpX-like, mitochondrial [*Bombyx mori*]
LOC101741131	1.372	1.11E-07	2.42E-08	XP_004934299.1| 2.8e-82| voltage-dependent L-type calcium channel subunit beta-2-like, partial [*Bombyx mori*]
LOC101741156	–1.335	1.90E-95	7.67E-97	XP_004926607.1| 2.2e-293| synaptic vesicle 2-related protein isoform X1 [*Bombyx mori*]
LOC101741524	–1.214	0	0	XP_004926609.1| 9.4e-297| synaptic vesicle glycoprotein 2C [*Bombyx mori*]
LOC101741700	–1.279	3.27E-07	7.40E-08	XP_004922728.1| 1.3e-163| sarcoplasmic calcium-binding protein [*Bombyx mori*]
LOC101741853	–1.476	5.92E-08	1.28E-08	XP_004923628.1| 0.0e+00| potassium channel subfamily K member 9 [*Bombyx mori*]
LOC101742903	–9.216	0	0	XP_004931132.1| 2.9e-298| synaptic vesicle glycoprotein 2B isoform X2 [*Bombyx mori*]
LOC101743453	1.076	1.85E-05	4.99E-06	XP_012545879.1| 0.0e+00| solute carrier organic anion transporter family member 4A1 isoform X1 [*Bombyx mori*]
LOC101743804	–2	4.54E-05	1.28E-05	XP_004932640.2| 0.0e+00| glutamate receptor ionotropic, kainate 2 [*Bombyx mori*]
LOC101744119	1.924	1.97E-21	2.28E-22	XP_012545589.1| 9.2e-306| putative inorganic phosphate cotransporter [*Bombyx mori*]
LOC101744132	2.585	1.93E-10	3.49E-11	NP_001299580.1| 0.0e+00| potassium voltage-gated channel subfamily H member 6-like [*Bombyx mori*]
LOC101744427	4.260	0	0	XP_004930921.1| 9.6e-294| monocarboxylate transporter 12 [*Bombyx mori*]
LOC101744522	4.392	6.42E-47	4.41E-48	XP_012549165.1| 1.1e-262| monocarboxylate transporter 1 isoform X1 [*Bombyx mori*]
LOC101744568	1.313	2.53E-135	7.85E-137	XP_004929500.1| 2.9e-205| calcium/calmodulin-dependent protein kinase type 1 [*Bombyx mori*]
LOC101744605	–1.141	1.53E-25	1.57E-26	XP_004925299.1| 1.4e-287| synaptic vesicle glycoprotein 2B [*Bombyx mori*]
LOC101744652	–1.264	4.80E-157	1.30E-158	XP_012546196.1| 4.5e-299| synaptic vesicle glycoprotein 2A [*Bombyx mori*]
LOC101744748	–2.429	1.46E-06	3.54E-07	XP_012545105.1| 1.3e-148| synaptic vesicle glycoprotein 2C-like [*Bombyx mori*]
LOC101744857	1.546	1.67E-116	5.80E-118	XP_004929389.1| 1.9e-296| sodium/hydrogen exchanger 9B2 isoform X1 [*Bombyx mori*]
LOC101745137	7.099	5.44E-175	1.32E-176	XP_004927807.1| 7.2e-259| monocarboxylate transporter 7 [*Bombyx mori*]
LOC101745430	1.410	5.33E-13	8.45E-14	XP_012547869.1| 0.0e+00| regulating synaptic membrane exocytosis protein 2 isoform X1 [*Bombyx mori*]
LOC101745500	–1.183	0.000355605	0.000111834	XP_004933911.1| 4.4e-82| heat shock 70 kDa protein, partial [*Bombyx mori*]
LOC101745659	1.848	7.47E-11	1.32E-11	XP_012544306.1| 1.1e-246| potassium voltage-gated channel protein Shaw [*Bombyx mori*]
LOC101745823	1.359	1.90E-14	2.83E-15	XP_004927809.1| 1.3e-252| proton-coupled amino acid transporter-like protein pathetic [*Bombyx mori*]
LOC101745861	1.563	4.86E-14	7.34E-15	XP_004924747.1| 8.7e-277| proton-coupled folate transporter [*Bombyx mori*]
LOC101745863	2.007	1.09E-30	9.81E-32	XP_004926413.2| 0.0e+00| sodium-independent sulfate anion transporter isoform X1 [*Bombyx mori*]
LOC101746012	1.215	2.77E-107	1.03E-108	XP_004929151.1| 1.3e-193| sodium/potassium-transporting ATPase subunit beta-2 [*Bombyx mori*]
LOC101746043	3.937	1.26E-24	1.33E-25	XP_004925372.1| 4.0e-272| putative inorganic phosphate cotransporter [*Bombyx mori*]
LOC101746094	1.815	1.56E-73	7.68E-75	XP_004924179.1| 2.5e-232| 5-hydroxytryptamine receptor 1A isoform X1 [*Bombyx mori*]
LOC101746144	–4.023	2.64E-258	4.57E-260	XP_004927040.1| 0.0e+00| sodium-dependent nutrient amino acid transporter 1-like [*Bombyx mori*]
LOC101746226	1.413	1.30E-38	1.00E-39	NP_001296484.1| 0.0e+00| transient receptor potential channel pyrexia [*Bombyx mori*]
LOC101746636	2.115	0.000333338	0.000104462	XP_021206441.1| 0.0e+00| LOW QUALITY PROTEIN: glutamate receptor ionotropic, delta-2 [*Bombyx mori*]
LOC101746651	4.459	1.00E-09	1.90E-10	XP_004923028.1| 5.4e-188| probable uridine nucleosidase 2 [*Bombyx mori*]
LOC101746887	3.716	9.27E-06	2.42E-06	XP_004923030.1| 1.3e-188| probable uridine nucleosidase 2 isoform X2 [*Bombyx mori*]
LOC101747040	–1.322	2.92E-11	5.04E-12	XP_004934379.2| 5.0e-69| proton-coupled amino acid transporter-like protein pathetic [*Bombyx mori*]
LOC101747147	3.306	6.38E-26	6.44E-27	XP_004932959.1| 0.0e+00| sodium-coupled monocarboxylate transporter 2 [*Bombyx mori*]
LOC101747179	–3.103	2.10E-229	4.05E-231	XP_012547428.1| 1.6e-283| proton-coupled amino acid transporter-like protein pathetic [*Bombyx mori*]
LOC105841243	1.476	1.09E-17	1.41E-18	XP_012548721.1| 3.1e-175| ATP-binding cassette sub-family G member 1-like [*Bombyx mori*]
LOC105841453	–2.195	0	0	XP_012544828.1| 1.3e-263| proton-coupled amino acid transporter 1 [*Bombyx mori*]
LOC105841494	6.687	0.000224041	6.86E-05	XP_012545113.2| 6.9e-53| synaptic vesicle glycoprotein 2B [*Bombyx mori*]
LOC105841866	–1.117	1.75E-12	2.85E-13	XP_021204603.1| 7.2e-157| glutamate receptor ionotropic, kainate 2-like [*Bombyx mori*]
Pi-tl	1.241	1.94E-22	2.18E-23	NP_001036907.1| 7.7e-205| inorganic phosphate transporter 1 [*Bombyx mori*]
Hsp23.7	–3.115	1.81E-167	4.64E-169	NP_001036942.1| 4.0e-115| heat shock protein hsp23.7 precursor [*Bombyx mori*]
Kmo	5.373	1.49E-101	5.80E-103	NP_001106135.1| 6.6e-271| kynurenine 3-monooxygenase [*Bombyx mori*]
Ser-4	2.322	1.86E-08	3.88E-09	NP_001037502.1| 1.1e-251| 5-hydroxytryptamine receptor [*Bombyx mori*]
Adh	5.381	1.95E-09	3.79E-10	NP_001037610.1| 1.9e-147| putative alcohol dehydrogenase [*Bombyx mori*]

In *D. melanogaster* tubules, as in vertebrate epithelia, convergence of distinct but interacting signal transduction pathways (Ca^2+^/NO/cGMP) stimulates calcium entry into principal cells via several classes of calcium channels, resulting in increased fluid transport (Choi et al., 1995; Davies, 2000). In this study, we examined whether the NOS pathway could be influenced after *BNGR-A2* mutation. In mutant animals, the transcriptional level of *NOS2* was significantly up-regulated by 143% (Figure 4C), NOS enzyme activity increased by 15.39% (Figure 4D), and NO concentration increased by 6.85% (Figure 4E), as compared with WT animals. These results supported that the NOS pathway was accelerated in mutant animals.

### Insulin and Ecdysone Signaling Pathways in *BNGR-A2* Mutants

**FIGURE 5:**
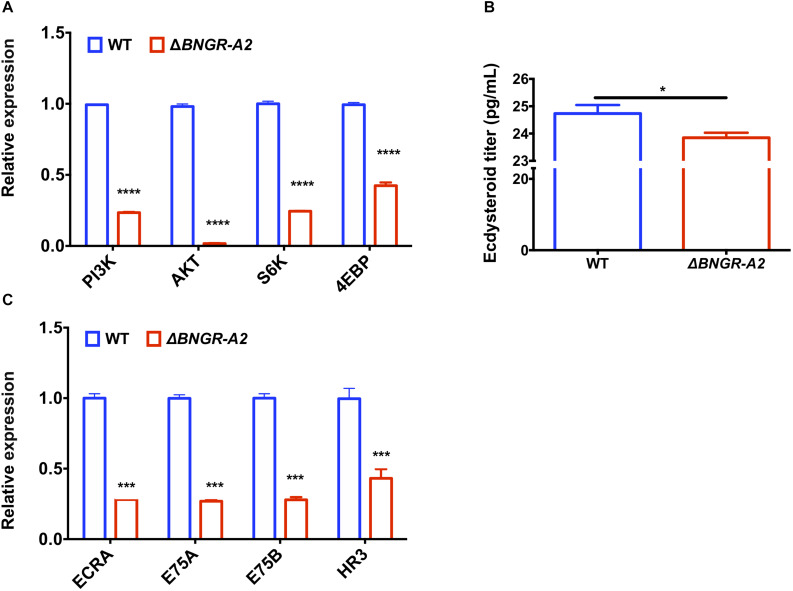
*BNGR-A2* regulates transcriptional levels of genes in insulin signaling pathway and 20E signaling pathway. **(A)** Change in expression of genes in insulin signaling pathway in fat body (FB). **(B)** Change in expression of ecdysone responsive genes in fat body (FB). The data show mean ± SEM (*n* = 3). **(C)** Relative ecdysteriod titers in the hemolymph at L5D3 stage of WT and △*BNGR-A2* animals. Hemolymph was collected from five larvae, and the pooled samples were used to determine ecdysteriod titers.

To further investigate the molecular mechanisms underlying phenotypic defects in the growth of *BNGR-A2* animals, transcription levels of key genes in the insulin and ecdysone signaling pathways were investigated in L5D3 larval fat body of WT and *BNGR-A2* mutants. Relative mRNA expressions of key genes in insulin signaling pathways including *PI3K*, *AKT*, *S6K*, and *4EBP* were down-regulated, suggesting that knockout of *BNGR-A2* affected the insulin signaling pathway (Figure 5A). Lower ecdysteroid titers and downregulation of 20E-related key genes, including ECRA, E75A, E75B and HR3, suggested that the 20E signaling pathway was affected after *BNGR-A2* disruption (Figures 5B,C).

## Discussion

**FIGURE 6:**
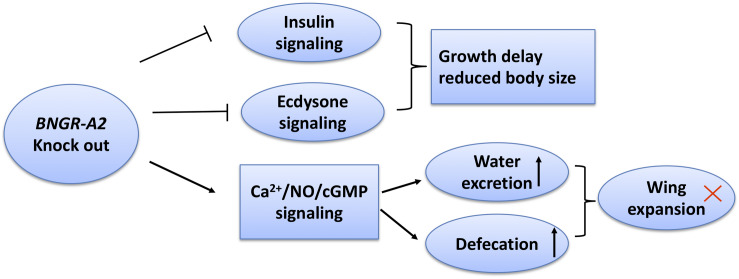
Scheme summarizing the roles of *BNGR-A2* in *Bombyx mori*. In *BNGR-A2* knock-out silkworm larvae, insulin signaling is reduced and ecdysone signaling inhibition causes growth delay. Through the Ca^2+^/NO/cGMP signaling pathway, body water decreases and the rate of excretion increases. Eventually, the body pressure drops to critical levels and the adult wings fail to expand.

ITP belongs to the class of CHH family neuropeptides and has been functionally characterized in many insect species (Drexler et al., 2007; Dircksen et al., 2008; Webster et al., 2012; Nagai et al., 2014; Yu et al., 2016). However, little is known regarding ITP signaling pathways in insects, and no insect ITPRs have been identified thus far, except in *B. mori* (Nagai et al., 2014). Here, we comprehensively investigated physiological functions of *BNGR-A2* in *B. mori* by using CRISPR/Cas9-mediated gene disruption. The NOS enzyme activity, NO content, and calmodulin transcription levels were significantly higher when *BNGR-A2* was knocked out. The downstream Ca^2+^/NO/cGMP signaling pathways were activated, resulting in the loss of water in the body. The decrease in the water content and concurrent increase in the amount of excretion in the mutant animals resulted in insufficient pressure in the adult body after emergence and ultimately led to the failure of wing expansion (Figure 6). *BNGR-A2* deficiency affected silkworm growth and development by inhibiting ecdysone and insulin signaling. These results reveal the important role *BNGR-A2* plays in insect larval growth rate, wing expansion, and water homeostasis (Figure 6).

In the insect excretory system, the Malpighian tubules and hindgut are involved in water preservation and absorption, which aid in maintaining water balance, allowing the organism to tolerate external hypertonicity or dryness (Meredith et al., 1996). This process is strictly regulated by neuropeptides, including diuretic hormone 31 (DH31) (Coast et al., 2001), diuretic hormone 44 (DH44), leucokinin (Cannell et al., 2016), and capa (Terhzaz et al., 2015), as well as antidiuretic hormones such as ITP. In insects, the diuretic peptide acts as a scavenging hormone, promoting the formation of primary urine in malpighian tubules and inducing rapid circulation of hemolymph and frequent hypoperistalsis of the digestive tract. Diuretic peptides promote the entry of raw urine into the hindgut, aid in moving dry food residue through the intestine (Gäde, 2004). The antidiuretic hormone acts on the hindgut to promote water reabsorption and plays an important role in insect water retention (Gäde, 2004). In previous studies, the NO signaling pathway was reported to be involved in the preservation of water in Diptera (Davies, 2000). NO is produced from the amino acid L-arginine by the enzyme action of nitric oxide synthase (NOS). NOS is encoded by a large multigene family, and occurs several isoforms. NOS isoforms have been classified into either calcium-dependent or calcium-independent enzymes, and belong to constitutive (ecNOS and ncNOS) or inducible NOS (iNOS) (Davies, 2000). Liquid transport was found to be controlled by NOS in *Drosophila* Malpighian tubules (Davies, 2000). In the late stage of *Plasmodium berghei* infection of *A. stephensi* in Diptera, NOS activity was significantly increased, likely due to the increase of hemolymph clearance (Dimopoulos et al., 1998). The NOS isoform in mosquitoes also contains Ca^2+^/calmodulin binding sites, indicating that NOS activation is associated with a regulatory calcium signaling process (Davies, 2000). In *B. mori*, two types of NO synthase were found in the malpighian tube. During the gut purging phase of *B. mori* larvae, here considered the excretion phase, the activity of NOS in malpighian tubule was significantly increased (Choi et al., 1995). We also found NOS activity and NO content to be significantly higher in the mutant *B. mori* than in WT animals. Based on hindgut transcriptome and qPCR data, downstream Ca^2+^/NO/cGMP signaling pathways were activated and 46 DEGs were annotated in the pathways named above when *BNGR-A2* was knocked out (Table 2). The transcription levels of the DEGs in mutant *B. mori*, including those encoding aquaporins, ion cotransporters, calmodulin (CaM), and NOS, were significantly higher than those of the WT animals. This is the first work to show that knockout of *BNGR-A2* in silkworms caused a (1.42 ± 0.035)% reduction in body water. Previous research showed that in humans, 2% dehydration leads to diminished mental activity and cognitive ability (Grandjean and Grandjean, 2007). Our results revealed that the Ca^2+^/NO/cGMP signaling pathway was involved in the transportation of water in *B. mori*, indicating that *BNGR-A2* affects the resorption of the hindgut water.

*BNGR-A2* was also found to play a critical role in the development of *B. mori*. The mutant larvae had significantly lower body weights than WT larvae at the same stage. The mutants showed slower preadult development, and larval development was extended by 3 days. Although development was prolonged, the pupal body weight showed no significant difference between mutant and WT *B. mori*. The size of adults was primarily determined by two factors: duration of growth and growth rate. The ecdysone and juvenile hormones synergistically regulate insect developmental metamorphosis and determine the duration of insect growth. The growth and death signals, such as insulin, nutrition, and cell contact inhibition, as well as their conduction pathways, control cell division, growth, differentiation, death, and ultimately determine the insect’s growth rate (Colombani et al., 2005; OrmeSally and Leevers, 2005). We discovered that the ecdysone titer is lower and the ecdysone pathway genes were down-regulated in the Δ *BNGR-A2*. The key genes of the insulin signaling pathway in the mutant were down-regulated, and the results indicated that the mutation of *BNGR-A2* disrupted the expression of other genes in the insulin signaling pathway, affecting the nutritional signaling pathway of the silkworm. This ultimately led to changes in the duration of development of mutant silkworms. However, the regulatory interaction of *BNGR-A2* between water homeostasis and ecdysone, as well as the insulin signaling pathways, needs further investigation.

## Data Availability Statement

All datasets generated for this study are included in the article/Supplementary Material.

## Author Contributions

LS and AT designed the research and wrote the manuscript. ZZ, RZ, YY, and FY performed the experiments and analyzed the data. AT revised the manuscript. All authors listed have approved the manuscript for publication.

## Conflict of Interest

The authors declare that the research was conducted in the absence of any commercial or financial relationships that could be construed as a potential conflict of interest.

## Supplementary Material

FIGURE S1 Transcriptomic profiles influenced by *BNGR-A2*-loss in silkworm hindguts by RNA-seq. **(A)** M-versus-A plot, MA plot diagram of DEGs in the transcriptome. **(B)** Gene Ontology (GO) term of DEGs in the transcriptome. **(C)** Kyoto Encyclopedia of Genes and Genomes (KEGG) annotations of differentially expressed genes (DEGs) in the transcriptome.

TABLE S1 List of primers used in this study.
