# Supplementary figures and images for: Molecular Disruption of Ion Transport Peptide Receptor Results in Impaired Water Homeostasis and Developmental Defects in Bombyx mori
Source: Front Physiol. 2020 May 20;11:424. doi: 10.3389/fphys.2020.00424 (PMC7251169; doi:10.3389/fphys.2020.00424)

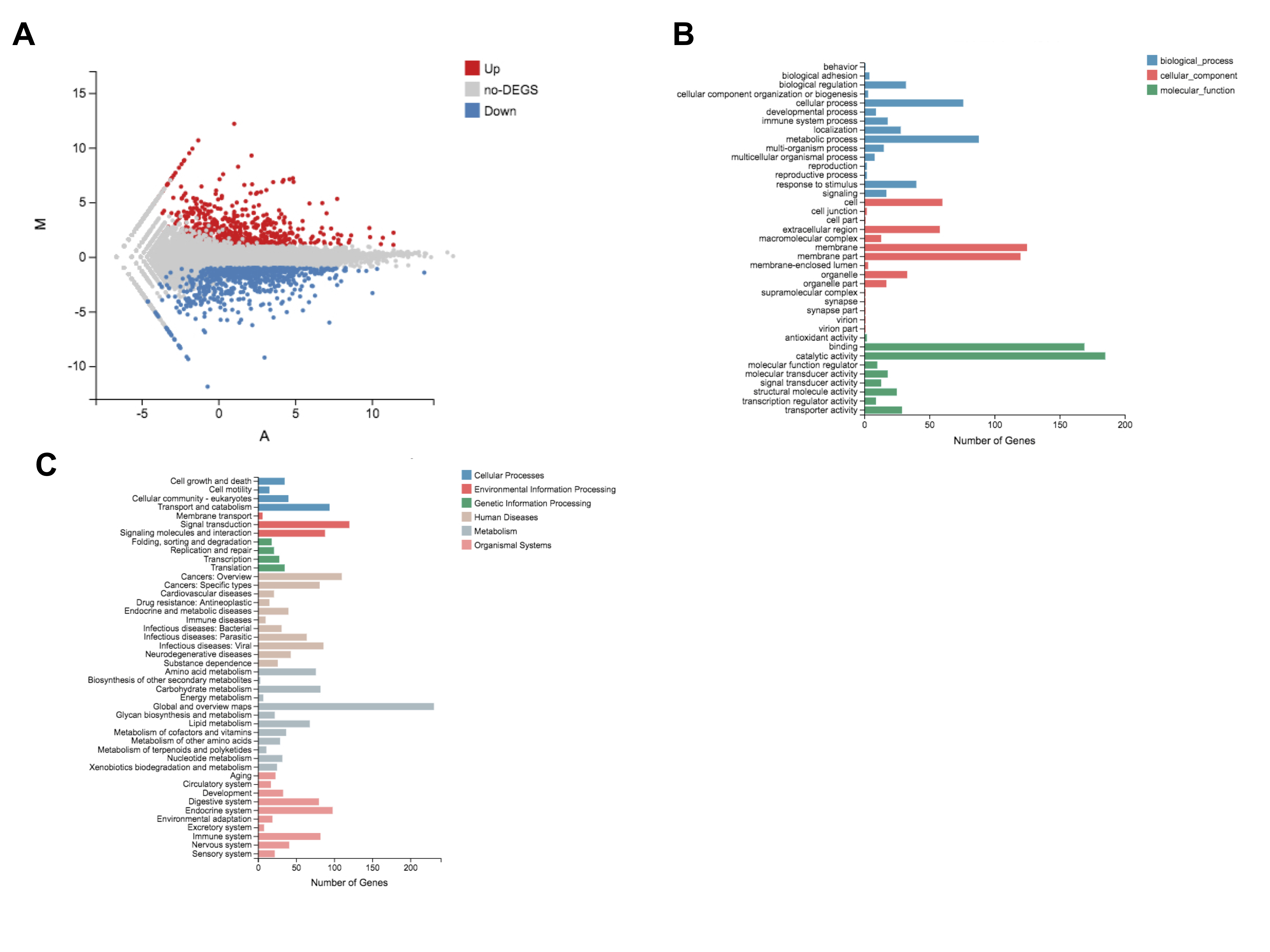

Supplement: FIGURE S1 — Transcriptomic profiles influenced by BNGR-A2-loss in silkworm hindguts by RNA-seq. (A) M-versus-A plot, MA plot diagram of DEGs in the transcriptome. (B) Gene Ontology (GO) term of DEGs in the transcriptome. (C) Kyoto Encyclopedia of Genes and Genomes (KEGG) annotations of differentially expressed genes (DEGs) in the transcriptome. [file Image_1.jpg]
