# Supplementary material for: Molecular Disruption of Ion Transport Peptide Receptor Results in Impaired Water Homeostasis and Developmental Defects in Bombyx mori
Source: Front Physiol. 2020 May 20;11:424. doi: 10.3389/fphys.2020.00424 (PMC7251169; doi:10.3389/fphys.2020.00424)
Supplement: TABLE S1 — List of primers used in this study. [file Table_1.pdf]

Table S1 List of primers used in this study.

Primer name	Primer sequence (5′ to 3′)	Primer usage
U6-KpnI-F	CTCACTATAGGGCGAATTGGAGGTTATGTAGTACACATTGTTGTA	Vector construction
A2-sgRNA1-R1	GGTCTTAGAAGATAGCCTCCACTTGTAGAGCACGATATTTTGTAT	
A2-sgRNA1-F1	GGAGGCTATCTTCTAAGACCGTTTTAGAGCTAGAAATAGCAAGTT	
Overlap-R	GCTAGCCATTGACTCCGCGGAGGTTATGTAGTACACATTGTTGTA	
Overlap-F	TTTTCTTGTTATAGATATCAAAAAAAGCACCGACTCGGTG	
A2-sgRNA2-R2	TAATTCGCGTGTCTTCAAACACTTGTAGAGCACGATATTTTGTAT	
A2-sgRNA2-F2	GTTTGAAGACACGCGAATTAGTTTTAGAGCTAGAAATAGCAAGTT	
U6-HidIII-R	CCGCGGAGTCAATGGCTAGCAAAAAAGCACCGACTCGGTG	
A2JC-F	CGGATAGTACTTTCTACGCGT	Mutation detection
A2JC-R	TCCATTGCTGGCGGGAC	
E75A-F	ATGTCTCCGGATAGTAGCTACGGGC	RT-qPCR
E75A-R	TATGCAATTCAGGTTCACGT	
E75B-F	ATGGTGCGAACCATGTCGTGTG	
E75B-R	GCGGTCACAGCTACATTTAGTA	
ECRA-F	TACAACGCACTGACGTGTGA	
ECRA-R	CACATTCAGGCCTCATTCCT	
HR3-F	AACTACCAGTGTCCTCGCAAC	

HR3-F	CCTTCTCTCGCTGCTTCTTC
PI3K-F	CTAGAAGCTGCGGAGTGGTA
PI3K-R	ACGGAAGCCATATCGACCTT
AKT-F	TCATGAACCACCCCTTCTTC
AKT-R	GCGAGGCTGTTCTCATTCTC
S6K-F	TGTGGAGGCAGAAGGATCAG
S6K-R	TCAGCCTTGGTATGTGCAGT
FOXO-F	GGGAACAATGGCAGAGTTGG
FOXO-R	CGTACGAAAGATTGCCCCAG
4EBP-F	CAATCTCCGATTTCCCAAAC
4EBP-R	CTGCGACTCGTCGAATGATA
Ugt-F	TTGGCTCAACGATCTGGAGT
Ugt-R	ACGCGTCTCCTTTTGTTGAC
ManI-F	TCACCCCTTCCAGATGACAC
ManI-R	AAAGCTTGGCCTTCTTGAGC
AQP-F	GGCTTTATAAAGCTCGACA
AQP-R	ATAGAGCAATGTGAATGGC
Ser-4-F	GATTAGTTGATGCATTGAGCC
Ser-4-R	CAGGTACAGCGCTTCCC
101739720-F	TGGGAGAGAAGAGAGCTTGT
101739720-R	ACTCCATGCACCTCAAACTC
Kymu-F	ACACCACTTATCAAGACGTTTATG

Kymu-R	CTTCGTACTCTTTTCACTCGTAAC
Kmo-F	GTTGAGAGAAGTCGGTTTAGAAG
Kmo-R	CCTTTTCTCAAATTTGATGCTATC
101740318-F	ATCATGGCTTTACCACTGG
101740318-R	CTTCCGTTATTATATGTTTCACCG
101747147-F	CAATATCGCCGAGTATTGCC
101747147-R	GATGCTGGTTATGTGACTTGC
101744132-F	ACGTGCCGTTGATCGTGA
101744132-R	CTGGTAGTCCGTTGAGACC
101745659-F	GGCGGTATAAGATACGAGAC
101745659-R	AAAGAGTGGACCGCACACA
101736988-F	TTTTTACGGATCGCGTGG
101736988-R	TGGCCGCTACAGCGTT
101737600-F	GACAAGTTAGCGTTTCTTCCGTTC
101737600-R	TGTGCTCACGAAGTATCTTATGT
NOS2-F	TTTCTTGGCACTTTCTAGGGAAG
NOS2-R	GTGGTATCTGTTTTCATCCATC
RP49-F	TCAATCGGATCGCTATGACA
RP49-R	ATGACGGGTCTTCTTGTTGG
